# Twenty-fourth Annual Meeting of the American Medical Association

**Published:** 1873-05

**Authors:** 


### Editorial.

### Twenty-fourth Annual Meeting of the American Medical Association.

The annual session of the Association opened at St. Louis, on the 6th inst., in Masonic Hall, there being present about four hundred members. The meeting was called to order by Dr. D. W. Yandell, of Louisville, the retiring President. After prayer had been made, Dr. John S. Moore, on behalf of the profession of St. Louis, gave an address of welcome, when the President elect, Dr. Thomas M. Logan, of Sacramento, Cal., took the Chair. Dr. J. B. Johnson, Chairman of the Committee of Arrangements, presented his report, and said that the members were invited, that evening, to attend a musical soiree; the next evening, a levee at the house of Col. J. D. Morrison; on the evening of the 8th, a lecture by Dr. J. J. Woodward, of Washington; on Friday afternoon, an excursion in carriages to Tower Grove Park and other points of interest. The Committee on Ethics, with Dr. N. S. Davis, Chairman, was appointed to decide as to the admission of gentlemen whose title to seats had been protested.

The President, Dr. Logan, then delivered an able address, encouraging the demand for a high standard of preliminary attainment in those entering the study of medicine, and congratulating the profession upon the measures adopted by Harvard College for the better qualification of its medical graduates. The tendency of the science of medicine in its progress of late is not to nihilism and expectancy merely, to the enlightened mind of the scientific physician it is towards exactitude and completeness. According to the advance in this direction is the value of the physician's aid enhanced. Respecting the influence or want of influence the profession has among the public, he says: "Just so long as society is ignorant of the knowledge which will enable it to rightly judge of the fitness of a profession to its wants, so long will there be impertinent interferences and quackery. Especially is this so in a country like ours, where every one is left to exercise their own judgment and choice. But let the people fully comprehend the laws by virtue of which they live, move and have being, and there will be no danger of their tampering with their highest earthly interests. Now it is just here to this point that I desire particularly to call your attention. It has been conceded that one of the greatest wants of the profession is some suitable and adequate means of communication between itself and the people. The science of hygiene is not above the people but for them. Let us throw away all puerile notions of the dignity of our calling and approach the people through the only channels by which they can be reached—the newspaper and the lecture-room. This is our work for the future—to educate the people. Too long for our interest and that of our race have medical men ignored this important duty. With dull apathy we have seen most other professions seeking to avail themselves of this power. When I recommend as one of the means of widening the usefulness of the Association the judicious instruction of the community in the science of life, I do not wish it to be understood that we are to do more than spread abroad such sound ideas of enlightened hygiene as will enable the people to co-operate with us in correcting all those formidable obliquities which are insiduously polluting the stream of humanity; so that our race may move onward and upward in purity of type to a higher and nobler manhood. In furtherance of this end I believe our Association will exert a powerful influence." In conclusion he suggested the holding of biennial meetings at Washington, and alternate ones at different points throughout the country.

A committee for the nomination of officers was appointed, consisting of one representative from each State. A communication from Dr. S. D. Gross, of Philadelphia, suggested that action be taken on an amendment to the Constitution proposed last year, that instead of a report on medical education, on medical literature, and climatology and epidemic diseases, there shall be annually delivered before the Association at its general meetings, an address in medicine, an address in surgery, an address in midwifery or the diseases of children. Dr. Woodward of Washington, presented a communication from

John M. Cuyler and nineteen other army surgeons, requesting such action as would assist in placing the medical gentlemen of the army on an equal footing with those of the navy, as well as with the officers of other staff corps of the army; also the admission of the young physicians of the country to the ranks of the army medical staff. A committee of five were appointed to memoralize Congress upon this subject, consisting of Drs. Kellar of Louisville, Askew of Delaware, Toner of Washington, Murphy of Cincinnati, and Davis of Chicago.

Dr. Carson, of Ohio, read a report of a committee upom medical education. The committee, recognizing the defects in medical education, were opposed to schemes to place the interests of that education under the control of the Government. There are limitations on medical education which are beyond the power of the profession or that Association to control to any great degree, but there is much in relation to the methods of medical teaching which is largely within the personal efforts of members of the profession to control. While not responsible for failure in the first direction, physicians are in the latter to a great degree. The remedy relied upon was personal efforts of members, and discussion before the Association, and not endeavoring to exercise legislative functions towards teaching bodies. The report was referred to the committee on publications.

A report of Dr. Yandell, Sr, of Louisuille, on medical literature was read. It referred with pride to the progress of this literature in this country, which is such as to inspire high hopes and to render medical students independent of foreign authors.

No essay prize was awarded this year. The Secretary read a list of names of those who had been appointed to represent the American Medical Association to the British Medical Association, and announced that the commission of the delegates would be made out immediately. The following named gentlemen compose the list: Drs. F. G. Smith, C. Wistar, J. S. Cohen of Philadelphia; Dr. E. Warren of Baltimore; Dr. C. L. Ives of New Haven; Dr. Edward Montgomery of St. Louis; and Drs. F. Barker, E. Segum and J. C. Hutchinson of New York

Upon recommendation of the committee on nominations, Dr. Johnson of St. Louis, chairman, the following officers were elected: President, Dr. J. M. Toner, District of Columbia; Vice Presidents, Drs. W. Y. Gadbury, of Mississippi, J. M. Keller, of Kentucky, N. C. Husted, of New York, L. F. Warner-of Massachusetts; Treasurer, Dr. C. Wistar, of Pennsylvanta: Secretary, Dr, T. A. McGraw, of Michigan; Committee of Arrangements, Drs. Brodie, J. A. Brown, M. Stewart, J. F. Noyes, E. W. Jenks, H. F. Lyster, D. O. Farrard, and Eugené Smith, all of Detroit.

The Treasurer reported that the balance in hand was $496.76.

A number of reports were read and referred to appropriate committees. Among these was an interesting report on the hospitals of the United States.

A lengthy report by Surgeon Guyon, U. S. N., was referred to the committee on medical education.

It was stated in a report by Dr. Toner, of Washington, that the number of medical societies in the United States is 405, of medical hospitals 174, also there are 82 public hospitals, together treating annually 144,750 patients.

Dr. Frederick Horner, Jr., U. S. Navy, offered a resolution that the American Medical Association appoint a committee of one member from each of the original thirteen States of the Union, to report at the centennial celebration, on the medical, surgical and biographical literature of the period of 1776. As a tribute to Joseph Warren, Benjamin Rush, Arthur Lee, Gen. Hugh Mercer, and other noble and patriotic physicians who aided to secure American independence, the resolution was adopted.

On motion of Dr. Peck, of Iowa, it was voted that the President and Secretary petition the next Congress, in behalf of the profession, for the issue of an edition of Volumes one and two of the first part of the "Medical and Surgical History of the War," such as to permit of its general distribution. It was voted that a committee be appointed to communicate with the Royal College of Physicians of London and request a representation of the Association in the first decennial revising of the "Nomenclature of Diseases." A resolution adopted on motion of Dr. Toner expressed the desirability of having an Inter national Medical Congress meet at the time of the celebration of the American Centennial to determine upon a uniform classification and nomenclature of diseases. An honorarium of $500 was voted to the permanent Secretary The appointment of members of the nominating committee from the thirteen original States was deferred till next meeting. Thanks were voted Col. Morrison, Henry Shaw and the citizens of St. Louis, also Dr. Woodward for his lecture. It was voted to employ stenographers and to publish each morning a report of the previous day's proceedings, during the sessions of the Association in the future. Dr. Logan, in his farewell address, mentioned the important and beneficial influence the Association had in California, and would have throughout the country hereafter. It was resolved to hold the next annual meeting at Detroit, commencing on the first Tuesday of June, 1873.

We understand that the meeting was a very pleasant one, and that the entertainments afforded were highly enjoyed. For our report of the session we are indebted to the St. Louis dailies and to the kindness of Dr. N. C. Husted, of New York, who was there present.

